# Network pharmacological analysis of corosolic acid reveals P4HA2 inhibits hepatocellular carcinoma progression

**DOI:** 10.1186/s12906-023-04008-6

**Published:** 2023-05-29

**Authors:** Fei-Feng Tang, Long Liu, Xiao-Ting Tian, Ning Li, Ying-Xiu Peng, Chun-Mei Qian, Ting-Ting Jia, Jing-Jin Liu, Wen-Hui Gao, Yan-Feng Xu

**Affiliations:** 1 Department of Pharmacy, Shanghai Municipal Hospital of Traditional Chinese Medicine, Shanghai University of Traditional Chinese Medicine, Shanghai, 200071 People’s Republic of China; 2 Department of Traditional Chinese Medicine, Tianyou Hospital of Tongji University, Shanghai, 200331 People’s Republic of China; 3 Shanghai Chest Hospital, Shanghai Institute of Thoracic Oncology, Shanghai Jiao Tong University School of Medicine, Shanghai, 200030 People’s Republic of China; 4 Central Laboratory, Shanghai Municipal Hospital of Traditional Chinese Medicine, Shanghai University of Traditional Chinese Medicine, Shanghai, 200071 People’s Republic of China

**Keywords:** Corosolic acid, Hepatocellular carcinoma, Network pharmacology, Proteomics, P4HA2

## Abstract

**Background:**

Corosolic acid is a pentacyclic triterpene acid with hypoglycemic, anti-inflammatory, and anti-cancer effects. However, its potential targets in hepatocellular carcinoma (HCC) are unknown, hindering clinical utilization.

**Methods:**

Differentially expressed proteins of the Bel-7404 cell line were identified with tandem mass tag analysis and differentially expressed genes (DEGs) of an HCC TCGA dataset using bioinformatics. Gene functions and pathways were inferred using the DAVID database. Online databases were used to establish P4HA2 expression in HCC (GEPIA2) and its relationship with patient survival (UALCAN and The Human Protein Atlas), the association between P4HA2 expression and immune cell infiltration (TIMER2), and DNA methylation of the P4HA2 gene (MethSurv). Cell proliferation, cell cycle, and cell death were assessed with PI and SYTOX-Green staining, CCK-8, and colony formation assays. Protein expression levels were detected by Western blotting.

**Results:**

A total of 44 differentially expressed proteins and 4498 DEGs were identified. Four genes whose proteins were also found in the differential protein profile but with opposing expressions were selected as candidate targets. The candidate gene prolyl 4-hydroxylase subunit alpha 2 (P4HA2) was recognized as the only potential target due to its high expression in public datasets, association with poor patient survival, and relation to immune cell infiltration in HCC tissues. Moreover, the DNA methylation status in 4 CpG islands of the P4HA2 gene correlated with a poor prognosis. Furthermore, corosolic acid treatment inhibited the proliferation of HCC cell lines Bel-7404 and HepG2 in a dose-dependent manner, caused G2/M phase cell cycle arrest, and promoted cell death. In addition, the treatment reduced P4HA2 protein levels.

**Conclusion:**

Our results indicate that P4HA2 is a potential target of corosolic acid. Thus, they contribute to understanding molecular changes in HCC after corosolic acid treatment and facilitate finding new treatment regimens.

**Supplementary Information:**

The online version contains supplementary material available at 10.1186/s12906-023-04008-6.

## Introduction

Primary liver cancer is the sixth most common malignant tumor worldwide, ranking fifth in the incidence rate and the third leading cause of cancer-related death in 2020. Its most frequent type is hepatocellular carcinoma (HCC), accounting for about 85–90% of primary liver cancers [1]. Systemic therapies for HCC are divided into 2 categories according to the mechanism of action [2]. The first is targeted therapy with multi-kinase inhibitors (MKI), such as sorafenib, lenvatinib, and regorafenib [3], and the second is immunotherapy with immunotherapeutic drugs, such as atezolizumab combined with bevacizumab or sintilimab combined with bevacizumab analogs [4, 5]. Multi-kinase inhibitor drugs usually do not cure hepatocellular carcinoma but only delay tumor progression, offering little survival benefit. Moreover, advanced tumors often find ways to escape target inhibition, causing drug resistance. Similarly, drug resistance and the low efficacy of single-agent checkpoint inhibitors mar the effectiveness of immunotherapy. Thus, current research directions explore finding combinations of immunotherapy with targeted or other therapies and developing new drugs for more effective liver cancer treatment.

Several traditional Chinese medicine preparations, including Huaier and cinobufacini combined with Jiedu, are approved adjuvant therapeutic agents after surgical resection for HCC in China [6, 7]. Other therapeutic agents, such as icaritin, β-elemene, and Ganfule, also have some benefits in treating advanced liver cancer [8–10]. Moreover, adjuvant chemotherapy based on traditional Chinese medicine is a new systemic treatment option for patients with HCC, given to improve the effectiveness of surgery or chemotherapy, reduce side effects, and prolong survival [11]. Corosolic acid is a pentacyclic triterpene acid extracted from plants such as crape myrtle (*Lagerstroemia speciosa*) and loquat (*Eriobotrya japonica*). It is attracting increasing attention since evidence suggests it inhibits the proliferation of various cancers, such as those in the colon, lungs, and stomach [12–14]. We previously showed that actinomycin D represses Yes1 associated transcriptional regulator (YAP1) protein, and its combination with corosolic acid increases its activity against liver cancer [15]. While corosolic acid has numerous anti-tumor effects in vivo, its mechanism of action is not understood, considerably limiting its clinical application. Therefore, elucidating how corosolic acid enhances chemosensitivity and predicting novel molecular targets are necessary for improving tumor treatment.

**Fig. 1:**
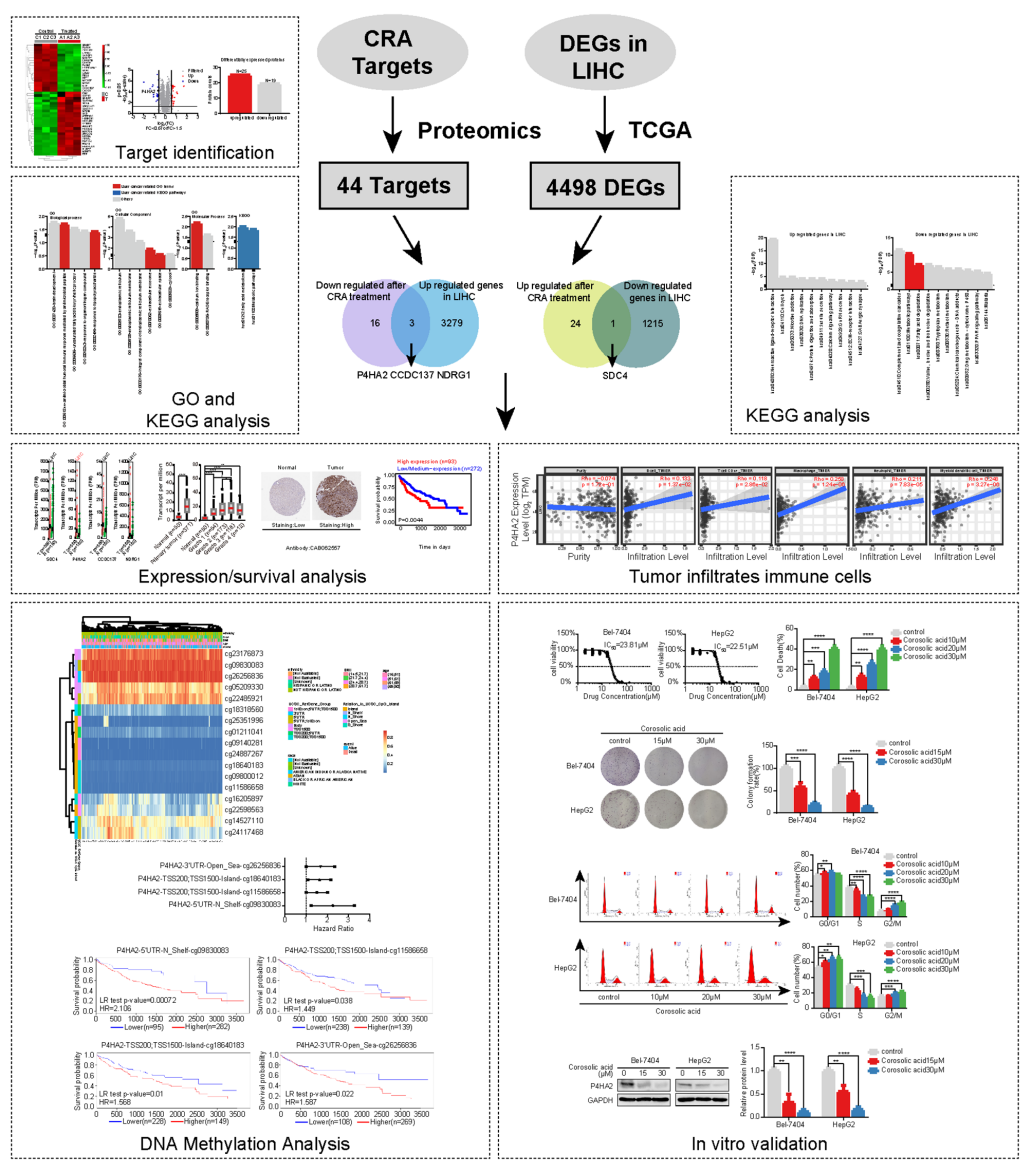
The workflow for the anti-HCC study of Corosolic acid (CRA) via a network pharmacological approach

Network pharmacology and bioinformatics analysis are tools that help discover the mechanisms of natural products [16, 17]. Furthermore, integrated bioinformatics analysis and proteomics identify differential proteins after cancer chemotherapy [18, 19]. Since the molecular targets of corosolic acid are unknown, we sought to identify the targets in this study by combining network pharmacology, bioinformatics, and proteomics. We discovered that corosolic acid targets prolyl 4-hydroxylase subunit alpha 2 (P4HA2) in HCC. We also analyzed the role of P4HA2 in immune cell infiltration, aberrant DNA methylation, and prognosis in this disease using bioinformatics to provide theoretical support for developing corosolic acid-based targeted therapies. The detailed technical strategy of this study is shown in Fig. 1.

## Materials and methods

### Cell culture

Hepatocellular carcinoma cell lines Bel-7404 and HepG2 were obtained from our previous study [20]. Mycoplasma contamination was excluded using short tandem repeat (STR) markers. The cells were cultured in DMEM (Hyclone, USA) supplemented with 10% fetal bovine serum (Gibco, USA) and 1% penicillin-streptomycin and maintained in a 37 °C incubator with 5% CO_2_.

### Drugs and immunoblotting antibodies

Corosolic acid was purchased from Solarbio, China. Cells were treated with corosolic acid at different concentration (0, 15, 30µM) for 24 h in immunoblotting. Primary antibodies for immunoblotting were P4HA2 (Proteintech Group, Inc; #13759-1-AP) and GAPDH (Cell Signaling Technology, Inc; #5174), diluted according to the manufacturer’s recommendation. Secondary antibodies were anti-rabbit IgG (Cell Signaling Technology, Inc; #7074) and anti-mouse IgG (Cell Signaling Technology, Inc; #7076) HRP-linked antibodies.

### Cell viability assay

Cell viability was detected using a Cell Counting Kit-8 (CCK-8) (Beyotime, China). Cells (8000 cells per well) were evenly seeded into 96-well plates and treated with corosolic acid at different concentration (2, 4, 8, 16, 32, 64, 128µM) for 24 h. The CCK-8 reagent and the medium were mixed into a working solution at a 1:10 ratio and used to incubate the cells for 1 to 3 h. The absorbance at 450 nm was detected using a microplate reader.

### Clone formation

Cells were evenly seeded into 6-well plates, and when visible cell clusters appeared, different concentrations of corosolic acid (0, 15, 30µM) were added for 24 h. Cells were washed twice with PBS, fixed with 4% paraformaldehyde, and stained with 0.1% crystal violet, followed by counting the clones per well.

### Flow cytometry analysis

The cell cycle was detected by a Cell Cycle Staining Kit (#70-CCS012; Multi Sciences Biotech, Co, Ltd, China) and analyzed by ModFit LT software version 3.1 (Verity.

Software House, USA). Cell death was quantified with SYTOX-Green staining (#KGA261, KeyGEN BioTECH, Co, Ltd, China), and the data were analyzed using FlowJo software version 10.8.1 (FlowJo, USA). The above experiments were treated with corosolic acid at different concentration (0, 10, 20, 30µM) for 24 h.

### Identification of differentially expressed proteins

Differential proteins of the Bel-7404 cell line before and after corosolic acid treatment were detected with tandem mass tags (TMT). The tag analysis was performed by Luming Biotechnology (China) [21]. The heat map showing differential protein expression was drawn by TB tools [22].

### Differential gene expression analysis

RNA sequencing data of liver cancer and normal tissues from The Cancer Genome Atlas (TCGA) database (https://portal.gdc.cancer.gov/) were downloaded from the UCSC XENA website (https://xenabrowser.net/datapages/, accessed on September 20, 2022). Differentially expressed genes (DEGs) between the tissues were identified using the R (v 4.2.1) package DESeq2 (v 1.36.0) by comparing gene expression data (HT-Seq counts) with a threshold of |log2 fold change| > 1 and adjusted *P* < 0.05 [23].

### GO and KEGG enrichment analysis

The Database for Annotation, Visualization and Integrated Discovery (DAVID) version 2021 (https://david.ncifcrf.gov/, accessed on September 25, 2022) [24] was used for GO and KEGG (https://www.kegg.jp/kegg/kegg1.html) [25–27] enrichment analyses of corosolic acid target molecules and DEGs in HCC. The significance level was set at *P* < 0.05.

### Generation of protein-protein interaction networks

Protein-protein interaction networks of corosolic acid targets in HCC were constructed with the STRING database (https://string-db.org/, accessed on September 25, 2022) [28]. The species was set to “Homo sapiens,” and interacting pairs with a combined score > 0.4 were extracted.

### GEPIA2

Gene Expression Profiling Interactive Analysis 2 (GEPIA2) (http://gepia2.cancer-pku.cn/, accessed on September 20, 2022) [29] is an online website containing gene expression data from TCGA database and Genotype-Tissue Expression (GTEx) project. It is used to identify gene expression in specific cancer. In this study, P4HA2 gene expression was assessed in liver hepatocellular carcinoma with a *P* < 0.05 threshold.

### UALCAN

The University of Alabama at Birmingham cancer (UALCAN) data analysis portal (http://ualcan.path.uab.edu/index.html, accessed on September 20, 2022) [30] is an exhaustive and interactive web resource for analyzing cancer omics data, allowing expression and survival analyses of hub genes in specific cancer. In this study, P4HA2 expression was investigated according to tumor grade, and the survival of patients with liver cancer was estimated in high and low P4HA2 expression groups.

### The human protein atlas

The Human Protein Atlas (https://www.proteinatlas.org/, accessed on October 1, 2022) [31] project is a website that provides information on the distribution of proteins across human tissues and cells. This database was used to establish the expression profile of the P4HA2 protein in liver cancer and normal tissues and explore its correlation with the survival of patients with liver cancer.

### TIMER2

Tumor Immune Estimation Resource 2.0 (TIMER2.0) (http://timer.cistrome.org, accessed on March 5, 2023) [32] is an extensive resource for analyses of tumor-infiltrating immune cells in cancer samples from TCGA database. It was utilized in this study to investigate the correlation between P4HA2 expression and tumor-infiltrating immune cells in liver hepatocellular carcinoma.

### MethSurv

MethSurv (https://biit.cs.ut.ee/methsurv/, accessed on March 5, 2023) [33] is a web tool that performs survival analysis based on CpG methylation patterns using methylation data of various human cancers. Epigenetic changes in the P4HA2 gene were assessed, and the relative DNA methylation site data were evaluated using this tool. The prognostic values of all methylation sites associated with P4HA2 were also determined.

### Statistical analysis

All data and error bars are expressed as the mean ± standard deviation of at least 3 independent experiments. Comparisons between multiple groups were calculated using one-way ANOVA. Data such as histograms and volcano plots were generated with data analysis and plotting in GraphPad Prism version 8.0.2 software. Differential gene expression was identified using the R (v 4.2.1) package DESeq2 (v 1.36.0). Statistical significance was concluded when *P* < 0.05.

## Results

### Establishing differential protein expression profile after corosolic acid treatment in hepatocellular carcinoma

**Fig. 2:**
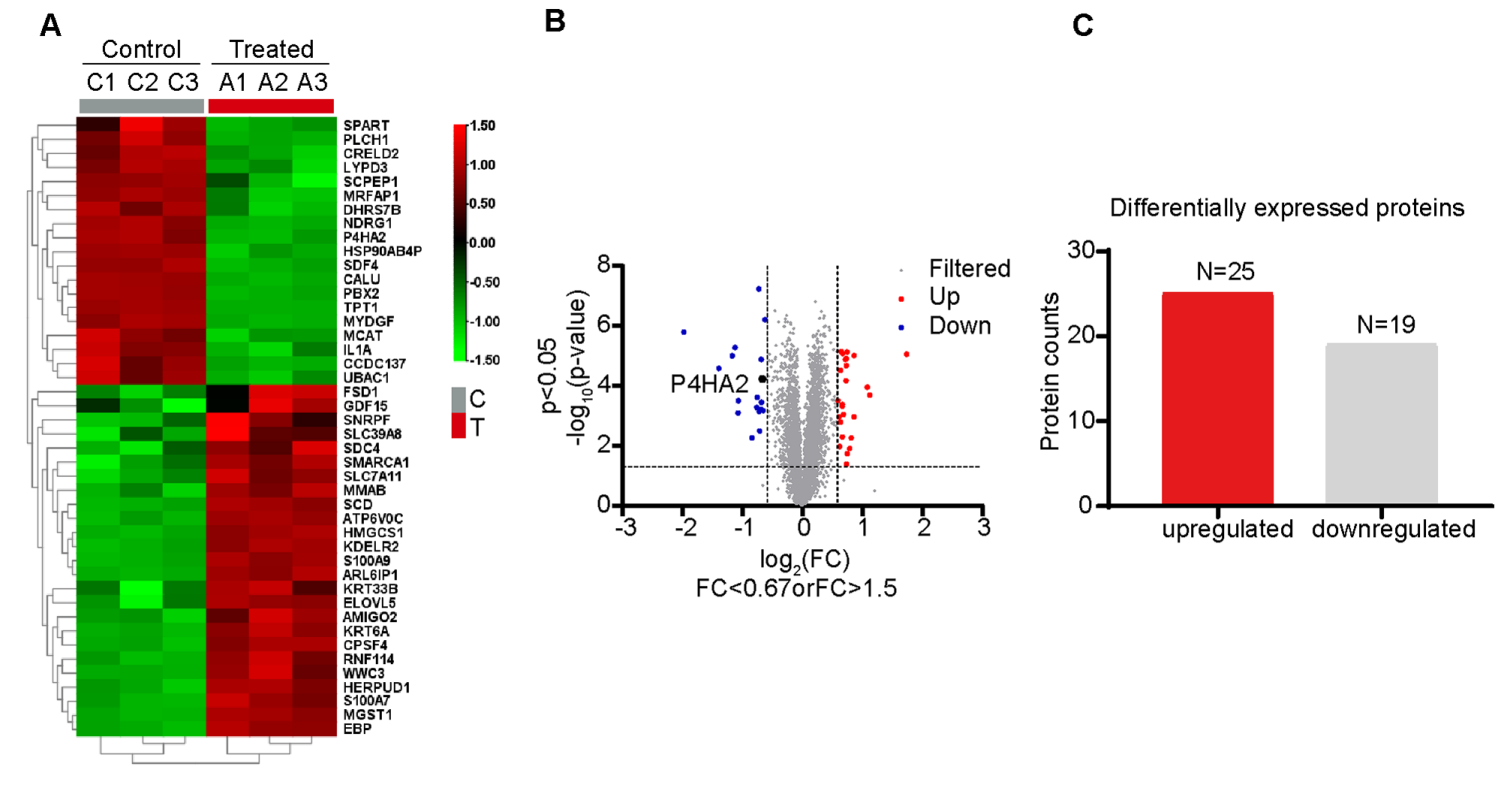
Identification of Differentially Expressed Proteins in Hepatocellular Carcinoma Cells Treated with Corosolic Acid. (**A**) Heat map showing 44 differentially expressed proteins between control (n = 3) and corosolic acid treated groups (n = 3), Rows are colored by the fold changes of the proteins in corosolic acid treated group relative to the corresponding proteins in control. (**B**) Volcano map Shows the differentially expressed proteins between control (n = 3) and corosolic acid treated groups (n = 3), Red indicates upregulation and blue indicates downregulation. (**C**) Histogram shows the proteins differentially expressed after corosolic acid treatment, with the number of up-regulated (red) and down-regulated (gray) proteins was displayed at the top of each bar

Because corosolic acid inhibits tumor growth, we sought to find the targets of corosolic acid in liver cancer cells. We treated HCC cell line Bel-7404 with corosolic acid and performed a tandem mass tag analysis to determine a differential protein expression profile. Differentially expressed proteins between the control and treatment groups were depicted as a heatmap and volcano map (Fig. 2A and B). We discovered by histogram statistics 44 differentially expressed proteins before and after corosolic acid treatment, of which 25 were up-regulated and 19 were down-regulated (Fig. 2C and Supplementary Table S1). A complete list of proteins recognized by mass tags is shown in Supplementary Table S1. We also constructed a protein-protein interaction network to detect possible interactors of the identified differential proteins and infer their molecular pathways (Supplementary Figure S1), in which P4HA2 does not interact with other proteins.

**Fig. 3:**
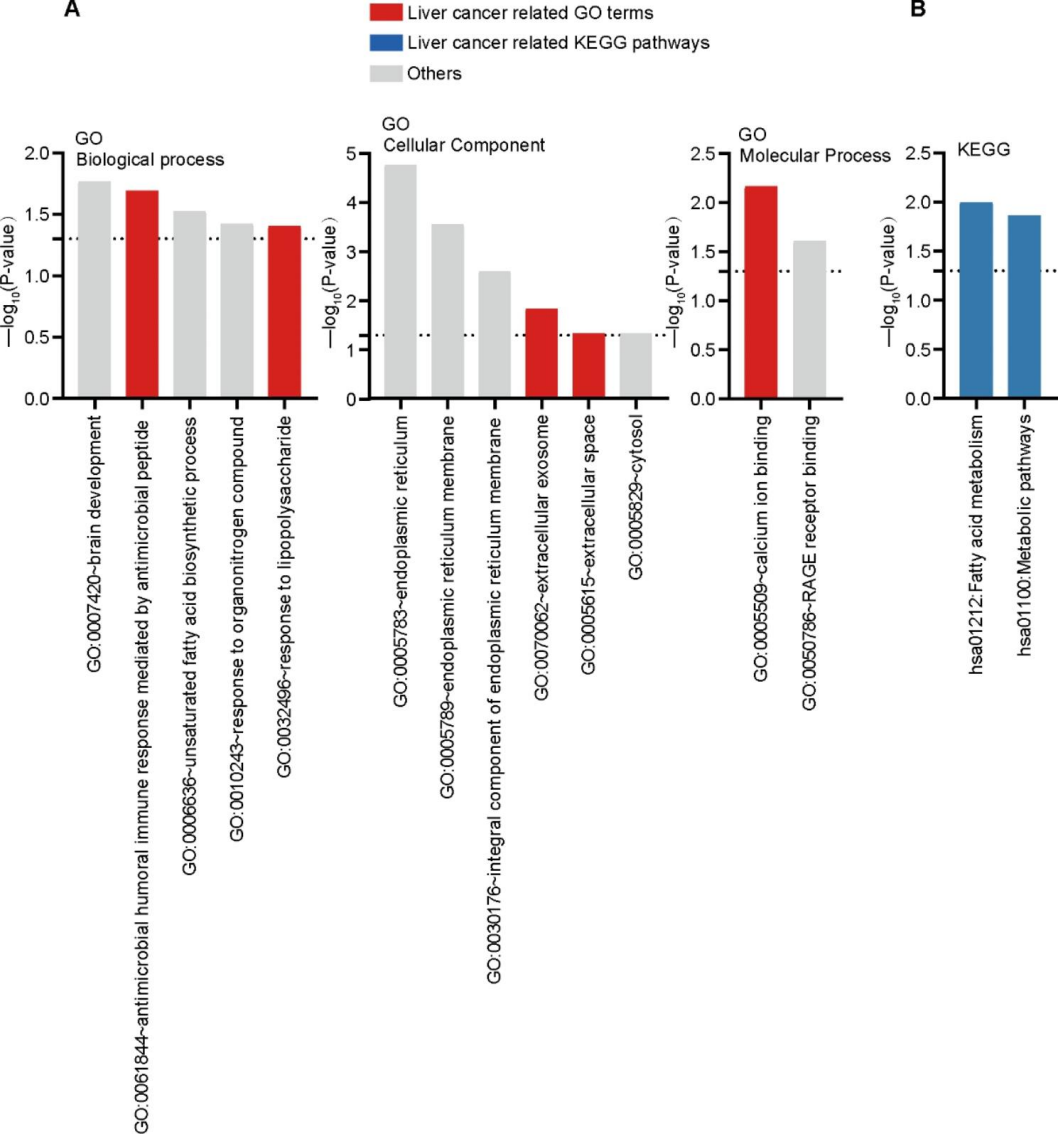
Bioinformatics analysis of differentially expressed proteins in hepatocellular carcinoma cells after corosolic acid treatment. (**A**) BP, CC, MF process analysis of 44 potential target proteins of corosolic acid, pathways that overlap with GO terms in liver cancer are shown in red. (**B**) KEGG analysis of 44 potential target proteins of corosolic acid, the pathways that overlap with the KEGG pathway in liver cancer are shown in blue

We performed a gene ontology (GO) functional annotation analysis to investigate the biological functions of the 44 differential proteins. We explored all 3 GO categories: biological processes (BP), cellular components (CC), and molecular functions (MF). In the BP category, the protein candidates were enriched with functions involved in brain development, antimicrobial humoral immune response mediated by antimicrobial peptide, unsaturated fatty acid biosynthetic process, response to organonitrogen compound, and response to lipopolysaccharide. Furthermore, in the CC category, they were enriched with locations such as the endoplasmic reticulum and its membrane, integral component of endoplasmic reticulum membrane, extracellular exosome, extracellular space, and cytosol. Finally, in the MF category, they were enriched with processes involving calcium ion and RAGE receptor bindings (Fig. 3A). We also wanted to know what pathways these proteins belong to and performed a pathway enrichment analysis. The enriched KEGG pathways (Fig. 3B) revealed that the differential proteins were mainly enriched in fatty acid metabolism and metabolic pathways.

### Determining differential gene expression profile in hepatocellular carcinoma

**Fig. 4:**
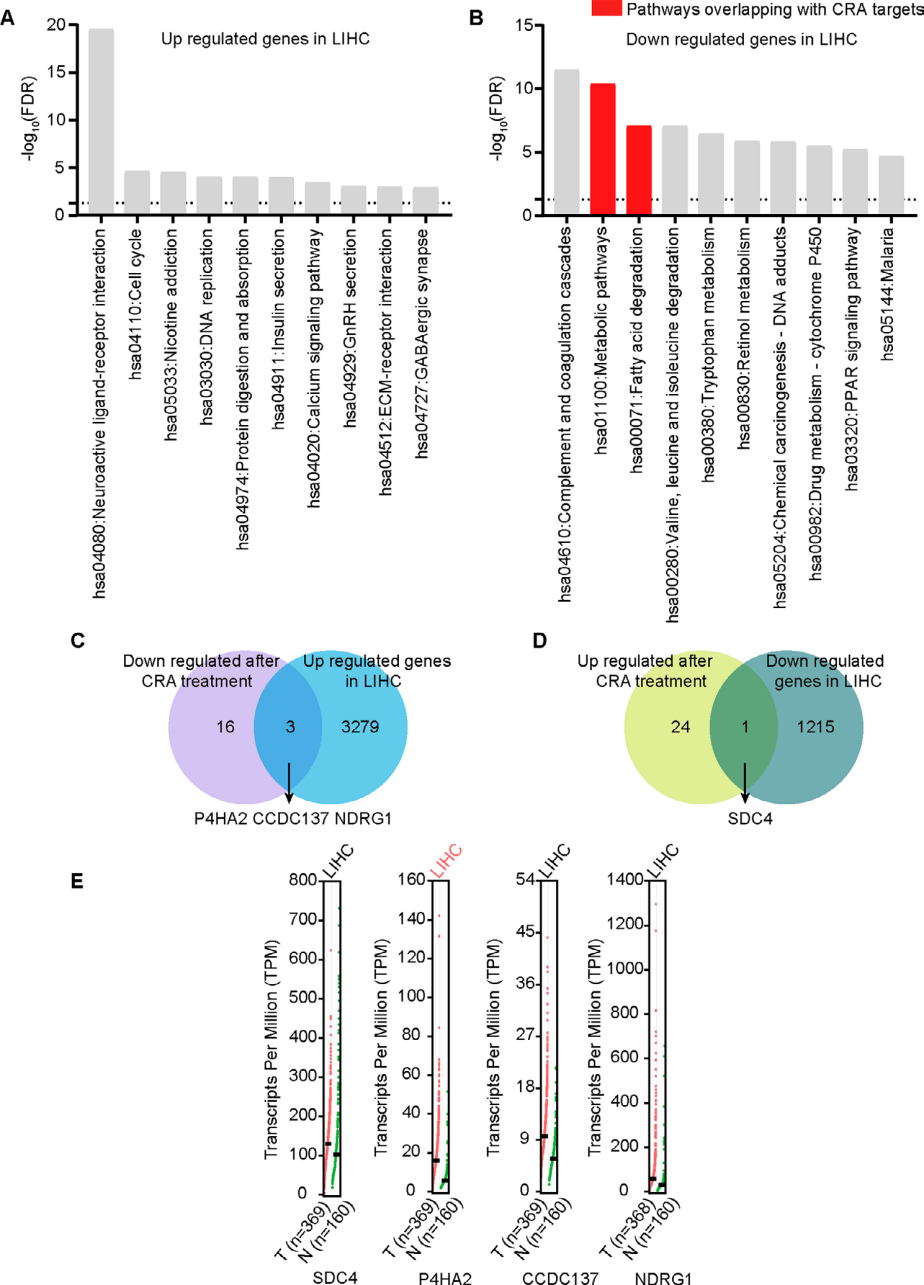
Differentially expressed genes between hepatocellular carcinoma and normal liver tissues. (**A**) Up-regulated KEGG pathway enrichment in LIHC. (**B**) Down-regulated KEGG pathway enrichment in LIHC, pathways overlapped with the corosolic acid targets were shown in red. (**C**) Venn diagram showed that the protein expression levels of P4HA2, CCDC137, and NDRG1 were down-regulated after corosolic acid treatment while their genes were up-regulated in hepatocellular carcinoma tissues. (**D**) Venn diagram showed that the protein expression levels of SDC4 was up-regulated after corosolic acid treatment while its gene was down-regulated in hepatocellular carcinoma tissues. (**E**) Comparison of mRNA Expression of P4HA2, CCDC137, NDRG1 and SDC4 in Hepatocellular Carcinoma and Normal Liver Tissues Using GEPIA Dataset, Including 369 cases of hepatocellular carcinoma and 160 cases of normal liver tissue

We analyzed differential gene expression in HCC cells to increase the chances of recognizing corosolic acid candidate targets. We used a publicly available TCGA dataset obtained by RNA sequencing of 369 liver cancer tissues and 50 paraneoplastic tissues and selected 4498 DEGs, of which 3282 were up-regulated and 1216 were down-regulated in cancer tissues compared with the paraneoplastic (Supplementary Table S2). Next, we did a pathway enrichment analysis using a *P* < 0.05 cut-off value to disclose the biological functions of these genes. We focused only on the top 10 enriched pathways. Up-regulated DEGs were most enriched in 3 KEGG pathways: neuroactive ligand-receptor interaction, cell cycle, and nicotine addiction (Fig. 4A). Conversely, down-regulated DEGs were most enriched in complement and coagulation cascades, metabolic pathways, and fatty acid degradation (Fig. 4B).

In summary, we identified 4498 DEGs in HCC, of which 3282 were up-regulated, while 1216 were down-regulated. We also discovered 44 differentially expressed proteins, of which 25 were up-regulated, whereas 19 were down-regulated. We looked at the intersection between 3282 genes up-regulated in HCC cells and 19 proteins down-regulated after the corosolic acid treatment. We found 3 candidate proteins down-regulated after the treatment whose genes were up-regulated in HCC tissues: P4HA2, coiled-coil domain containing 137 (CCDC137), and N-myc downstream regulated 1 (NDRG1). We also analyzed the intersection between 1216 genes down-regulated in HCC and 25 proteins up-regulated following the corosolic acid treatment and identified syndecan 4 (SDC4) as the only candidate protein. We presented the above analysis as Venn diagrams (Fig. 4C and D).

Since the TCGA dataset contained expression data of only 50 paraneoplastic tissues, we also used a dataset with 160 paraneoplastic tissues from the GEPIA2 database to verify the expression of the 4 candidates. After comparing the mRNA expression levels of P4HA2, CCDC137, NDRG1, and SDC4 between HCC and normal liver tissues, we observed that only the expression levels of P4HA2 were higher and significant in HCC tissues than in normal liver tissues (Fig. 4E), suggesting that P4HA2 is the only potential target of corosolic acid among the 4 candidates.

### High P4HA2 expression in hepatocellular carcinoma tissues correlates with poor prognosis

**Fig. 5:**
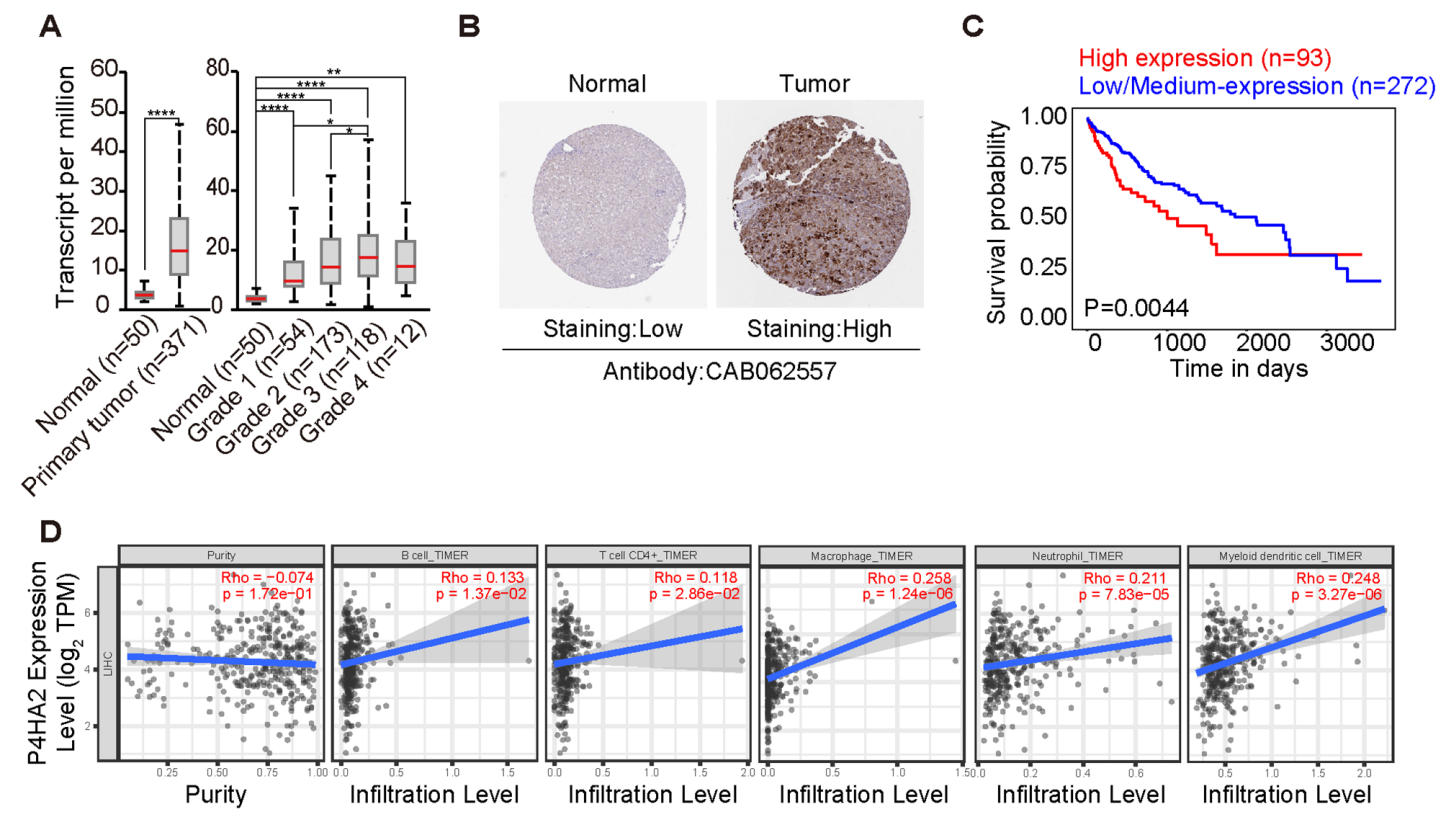
Upregulation of P4HA2 in hepatocellular carcinoma cells is closely associated with shorter cell survival time. (**A**) UALCAN database was used to detect mRNA expression levels of P4HA2 in normal liver tissues and hepatocellular carcinoma tissues, meanwhile showing mRNA expression levels of P4HA2 at different grades. (**B**) Immunohistochemical staining of P4HA2 in hepatocellular carcinoma and adjacent tissues in the HPA database, low means a low degree of staining, while high means a high degree of staining. (**C**) UALCAN database was used to detect the survival probability between patients with high and low / medium P4HA2 expression in hepatocellular carcinoma. (**D**) Correlation of P4HA2 expression with immune infiltration status in LIHC. *P < 0.05; **P < 0.01; ***P < 0.001; ****P < 0.001 indicates statistical significance

We used the UALCAN database to explore P4HA2 expression across different HCC grades. The expression of P4HA2 in HCC tissues was higher than in normal liver tissues, consistent with those in TCGA and GEPIA2 databases. Moreover, it was also higher in high-grade versus low-grade tumors (Fig. 5A). Low P4HA2 expression in normal controls but high in liver cancer tissues (Fig. 5B) was also observed in immunohistochemistry staining results retrieved from the Human Protein Atlas database, agreeing with those from the UALCAN database. We also investigated using the UALCAN database how P4HA2 expression affects the survival of patients with HCC. We discovered that patients with high P4HA2 expression had lower survival than those with low or medium P4HA2 expression (Fig. 5C). Kaplan-Meier method was used to evaluate the survival time data of P4HA2 gene in HPA database. The analysis results showed that higher levels of P4HA2 were related to shorter survival time (Supplementary Figure S2). Together, these results indicate that P4HA2 is up-regulated in HCC and is associated with poor patient prognosis.

The tumor microenvironment consists of fibroblasts, matrix, immune, and endothelial cells and is crucial for tumor occurrence and development. Therefore, we investigated the correlation between P4HA2 expression and the infiltration level of various immune cells in HCC. As shown in Fig. 5D, P4HA2 expression positively correlated with macrophages, neutrophils, B, CD4+, and dendritic cells, implying that P4HA2 is related to immune infiltration in HCC.

### Methylation status of P4HA2 gene in hepatocellular carcinoma correlates with prognosis

**Fig. 6:**
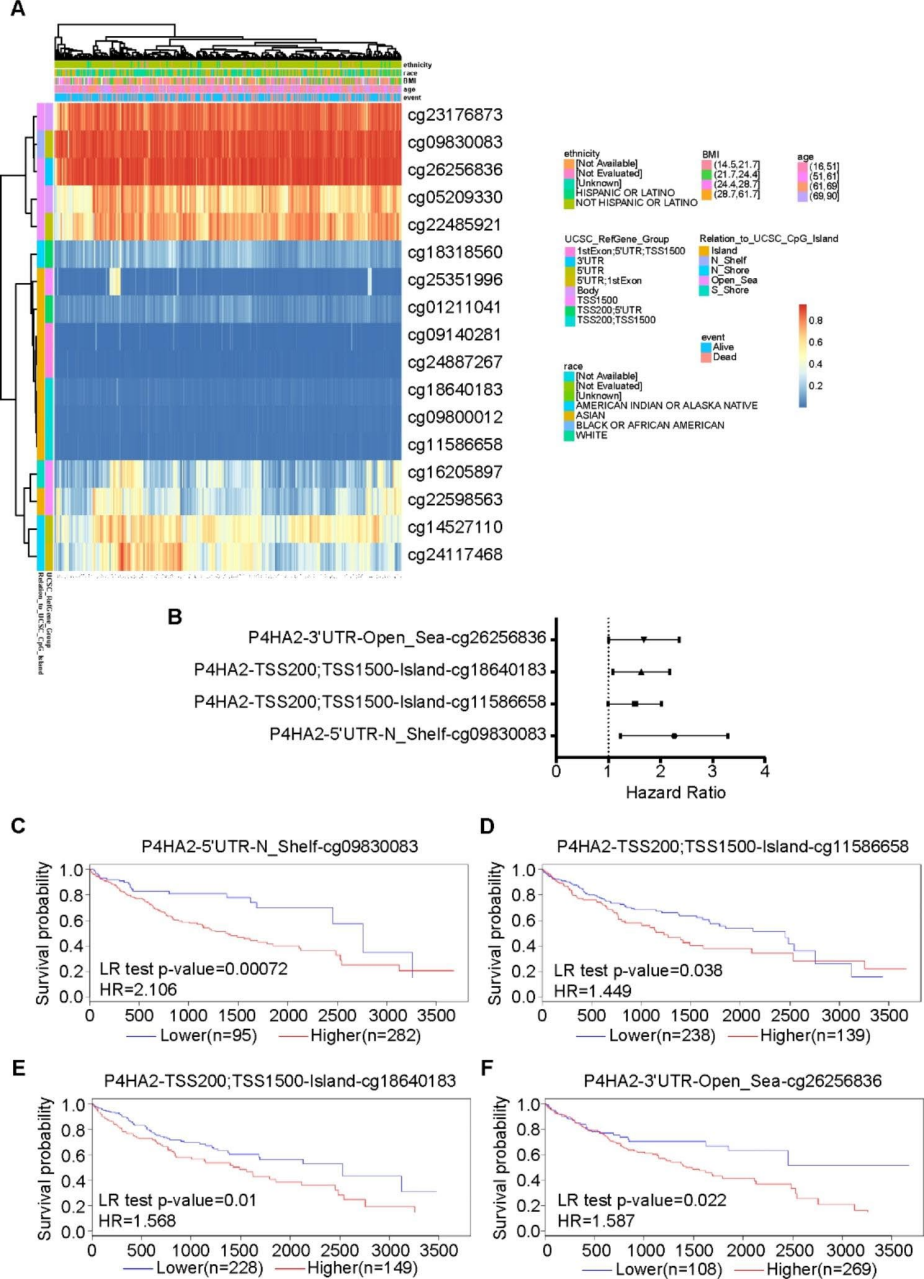
Methylation of P4HA2 was correlated with prognosis in liver cancer from MethSurv. (**A**) Heat map of methylation levels of the P4HA2 methylated probe. Red to blue scale indicates high to low expression. Various colorful side-boxes were used to represent the event, age, race, ethnicity, UCSC_refGene_Group, and relation to UCSC_CpG_island. (**B**) DNA methylation forest of P4HA2. (**C**) Association of methylation at 5’UTR-N_Shelf-cg09830083 in P4HA2 with patient OS. (**D**) Association of methylation at TSS200; TSS1500-Island-cg11586658 in P4HA2 with patient OS. (**E**) Association of methylation at TSS200; TSS1500-Island-cg18640183 in P4HA2 with patient OS. (**F**) Association of methylation at 3’UTR-Open_Sea-cg26256836 in P4HA2 with patient OS.

DNA methylation plays an essential role in regulating gene expression. Aberrant DNA methylation is one of the hallmarks of cancer development and correlates with clinical outcomes. We employed the MethSurv web tool to assess DNA methylation levels of the P4HA2 gene and the prognostic value of each CpG site using HCC methylation data from the TCGA database. We found that cg26256836 in the P4HA2 gene was the most methylated site (Fig. 6A) and cg09830083 (HR = 2.106; 95% CI, 1.32–3.362; *P* < 0.01) was the most powerful, locational risk factor (Fig. 6B and Supplementary Table S3). We also identified 4 CpGs within P4HA2 associated with poor prognosis of HCC: 5′ UTR-N_Shelf-cg09830083, TSS200; TSS1500-Island-cg11586658, TSS200; TSS1500-Island-cg18640183; and 3′ UTR-Open_Sea-cg26256836 (Fig. 6C-F).

#### Corosolic acid inhibits growth of hepatocellular carcinoma cells and decreases P4HA2 protein expression

**Fig. 7:**
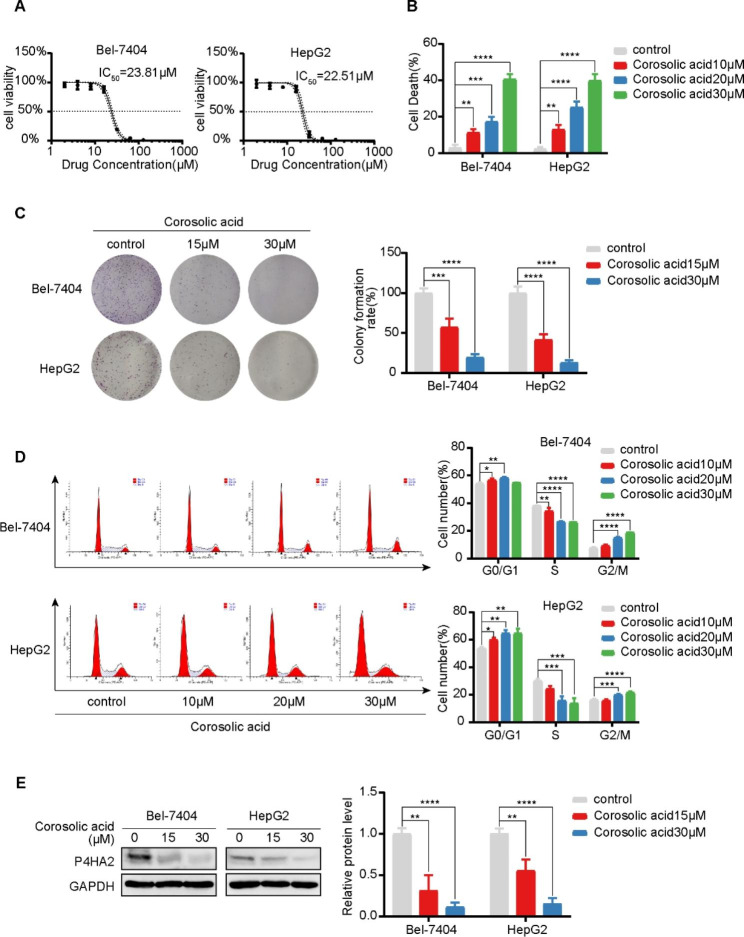
Effects of corosolic acid on various phenotypes of hepatocellular carcinoma cells (**A**) Hepatocellular carcinoma cells Bel-7404 and HepG2 were treated with different concentrations of corosolic acid (2, 4, 8, 16, 32, 64, 128µM) for 24 h, and CCK-8 assay was performed to detect the cytotoxicity of candidate drugs. IC_50_ values were calculated to evaluate the cytotoxicity of candidate drugs. (**B**) SYTOX-Green staining was used to detected Cell death after treatment with different concentrations of corosolic acid (0, 10, 20, 30µM) for 24 h. (**C**) Colony formation in Bel-7404 and HepG2 cells after 7 days, the colonies were treated with corosolic acid (0, 15, 30µM) for 24 h and then were fixed with 4% paraformaldehyde as well as stained with 0.1% crystal violet. (**D**) PI staining was used to detect the effect of corosolic acid (0, 10, 20, 30µM) for 24 h. Flow cytometry was used to detect the cell cycle distribution of Bel-7404 and HepG2 cells (**E**) Western blot was used to determine the expression level of P4HA2 protein in Bel-7404 and HepG2 cell lines after corosolic acid (0, 15, 30µM) treatment for 24 h. In order to save and recycle antibodies and save time, membranes were cut in horizontal strips at molecular weight ranges for target proteins and the information was shown in the Supplementary File. Data are shown as mean ± SD of three biological replicates. *P < 0.05; **P < 0.01; ***P < 0.001; ****P < 0.001 indicates statistical significance

We treated HCC cells Bel-7404 and HepG2 with increasing concentrations of corosolic acid to explore whether it affects cell growth. Both cell lines exhibited lower viability as acid concentrations rose and underwent more cell death in a dose-dependent manner (Fig. 7A and B). They also formed fewer colonies when exposed to the drug, and their numbers decreased in a dose-dependent way (Fig. 7C). Moreover, we also did a flow cytometry analysis to assess cell cycle distributions and found that corosolic acid induces G2/M phase block in these cells (Fig. 7D). These findings suggest that corosolic acid inhibits cell proliferation, promotes cell death, and induces G2/M phase arrest in HCC cells.

We quantified with Western blotting P4HA2 protein levels in the 2 HCC cell lines before and after a 24-h corosolic acid treatment to confirm that corosolic acid regulates P4HA2 in HCC. Indeed, the treatment reduced P4HA2 protein levels in both cell lines compared with the untreated conditions (Fig. 7E), supporting that P4HA2 is a potential target of corosolic acid in HCC.

## Discussion

Liver cancer remains a global health threat owing to its increasing incidence, diagnosis at an advanced stage, and few therapeutic choices for most patients [34]. Thus, novel, more effective treatments are necessary to fight the disease. Adjuvant chemotherapy based on traditional Chinese herbal medicine has become a novel therapeutic option for treating liver cancer in China. Of the notable compounds extracted from medicinal plants, corosolic acid has received much attention due to its anti-inflammatory and anti-diabetic activities and recently discovered anti-tumor effects. It induces apoptosis in lung adenocarcinoma cells by regulating the expression of anti-apoptotic proteins and in gastric cancer cells via the AMPK-mTOR pathway. It also induces caspase proteins, promoting the apoptosis of colon cancer cells [35–37]. Corosolic acid represses the growth of liver cancer cells in vitro and in vivo, and this inhibitory effect is enhanced when corosolic acid is combined with sorafenib [38]. Although some progress has been made, the knowledge about the mechanism of action of corosolic acid in HCC is still scant, demanding a lengthy process of uncovering potential targets of corosolic acid and unraveling its pharmacological mechanisms in HCC. The good news is that the recent advancements in bioinformatics have sped up identifying natural product targets and enabled the quicker discovery of new target molecules of corosolic acid through network pharmacology.

The HCC cell line Bel-7404 is multidrug resistant and highly malignant. In this study, we identified 44 differential proteins in this line before and after treating it with corosolic acid. These proteins were involved in specific cellular processes, such as brain development and antimicrobial humoral immune response mediated by antimicrobial peptide. We also analyzed the TCGA HCC dataset and identified 4498 differentially expressed genes. We selected those whose proteins responded to the corosolic acid treatment but in the opposite direction. We narrowed the search to 4 candidate genes: P4HA2, CCDC137, NDRG1, and SDC4 but focused only on P4HA2 as the potential target since the 3 remaining genes failed to show significant expression across different public datasets. Although the other 3 genes did not pass our selection criteria, they still may have a role in HCC development. For instance, CCDC137 is a biomarker for immune infiltration and poor prognosis of HCC [39]. The mRNA expression and DNA methylation of NDRG1 and its family members have implications in diagnosing HCC and estimating its prognosis [40]. In addition, SDC4 regulates angiogenesis and may be a therapeutic target for HCC [41].

The center of our study is P4HA2, a gene encoding a crucial enzyme involved in collagen formation [42]. Collagen is an essential component of the extracellular matrix [43], which can promote tumor development by initiating tumor cell proliferation, invasion, migration, epithelial-mesenchymal transition, and chemoresistance [44–46]. Abnormal P4HA2 expression indicates a poor outcome in liver, cervical, and ovarian cancers [47–49]. Aspirin represses P4HA2 expression through NF-κB and LMCD1-AS1/let-7 g pathways, inhibiting collagen deposition and preventing HCC progression [50]. Our study found that the P4HA2 protein was overexpressed in HCC tissues and correlated with a poor prognosis, agreeing with the previous studies.

With the development of immunotherapy as cancer therapy, tumor immunity has become the latest research hotspot. As core components of the tumor microenvironment, immune cells are decisive for the response to anti-tumor treatments and subsequent prognosis [51]. Our study also demonstrated a potential relationship between P4HA2 expression and tumor immune cell infiltration since P4HA2 expression showed a positive correlation with the levels of macrophages, neutrophils, B, CD4+, and dendritic cells in the HCC tissues.

Aberrant DNA methylation is a frequent epigenetic event in many human cancers, and the DNA methylation status of many genes is associated with cancer initiation and progression [52]. P4HA2 is a member of prolyl 4-hydroxylase family, the latter were detected down-regulated in almost all common B-cell lymphoma types and were associated with methylation of CpG islands in lymphomas and may be useful in differentiating disease subtypes [53]. We investigated the relationship between methylation levels in the P4HA2 gene and HCC prognosis and discovered hypermethylation of 4 CpG sites, cg09830083, cg11586658, cg18640183, and cg26256836, were associated with poor overall survival.

Additionally, we demonstrated that corosolic acid inhibits HCC cell proliferation and clone formation, induces cell cycle arrest, and decreases P4HA2 protein levels in vitro. These results suggest that corosolic acid represses the growth of HCC cells, underscoring its role in tumorigenesis. Importantly, given that corosolic acid represses the P4HA2 protein, we believe this protein is a vital target for treating HCC with corosolic acid.

In summary, this study demonstrates changes affecting HCC cells after exposure to corosolic acid and discloses P4HA2 as a potential target in corosolic acid anti-tumor process. These findings will facilitate the clinical application of corosolic acid and the identification of novel therapeutic targets. Admittedly, this study has some limitations since in vivo experiments that would verify our results are still missing, necessary to obtain a clearer picture of the complex underlying molecular mechanism of corosolic acid.

## Conclusion

This study found that P4HA2 is a potential target of corosolic acid and part of its anti-tumor mechanism. Its expression relates to the tumor infiltration status of multiple immune cells, suggesting P4HA2 has a decisive role in how patients with HCC respond to immunotherapy. Moreover, P4HA2 expression and methylation status are linked with HCC prognosis. Together, our findings contribute to understanding molecular changes in HCC after corosolic acid treatment, which is essential to finding new targets and designing effective chemotherapy regimens for future HCC treatments.

## Electronic supplementary material

Below is the link to the electronic supplementary material.

Supplementary Material 1

Supplementary Material 2

Supplementary Material 3

Supplementary Material 4

## Data Availability

A TCGA dataset containing gene expression data (named TCGA-LIHC.htseq_counts.tsv) was downloaded from the UCSC cancer browser (https://xenabrowser.net/datapages/). The original contributions presented in the study are included in the article/Supplementary Material. Further inquiries can be obtained from the corresponding authors.

## Abbreviations

HCC: Hepatocellular carcinoma
CRA: Corosolic acid
P4HA2: Prolyl 4-hydroxylase subunit alpha 2
CCDC137: Coiled-coil domain containing 137
NDRG1: N-myc downstream regulated 1
SDC4: Syndecan-4
DEGs: Differentially expressed genes
TMT: Tandem mass tags
PPI: Protein-protein interaction
ANOVA: One-way analysis of variance
FC: Fold change
GO: Gene ontology
BP: Biological process
CC: Cellular component
MF: Molecular function
KEGG: Kyoto encyclopedia of genes and genomes
LIHC: Liver hepatocellular carcinoma
TCGA: The Cancer Genome Atlas
HR: Hazard ratio
OS: Overall survival
TME: Tumor microenvironment
ECM: Extracellular matrix
EMT: Epithelial-mesenchymal transition.
