# Supplementary figures and images for: Network pharmacological analysis of corosolic acid reveals P4HA2 inhibits hepatocellular carcinoma progression
Source: BMC Complement Med Ther. 2023 May 29;23:171. doi: 10.1186/s12906-023-04008-6 (PMC10226252; doi:10.1186/s12906-023-04008-6)

**Figure 5E: Western blot original strips of P4HA2, GAPDH.**

**
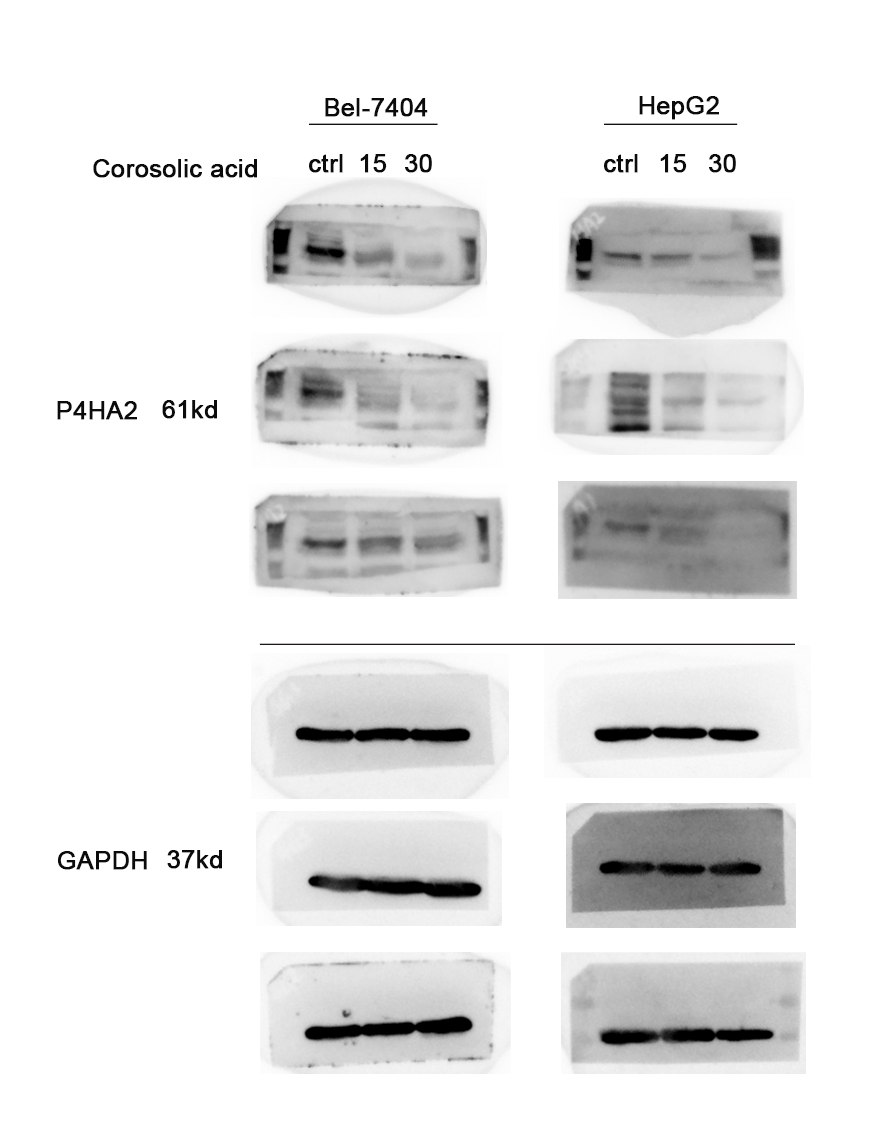
**

Supplement: Supplementary file 1 — Supplementary Material 1 [file 12906_2023_4008_MOESM1_ESM.docx]
