# Supplementary material for: Network pharmacological analysis of corosolic acid reveals P4HA2 inhibits hepatocellular carcinoma progression
Source: BMC Complement Med Ther. 2023 May 29;23:171. doi: 10.1186/s12906-023-04008-6 (PMC10226252; doi:10.1186/s12906-023-04008-6)
Supplement: Supplementary file 2 — Supplementary Material 2 [file 12906_2023_4008_MOESM2_ESM.pdf]

Bullet Edits

EDITORIAL CERTIFICATE

This document certifies that the paper listed below was edited and proofread for proper English language, grammar, punctuation, spelling, and overall style by one or more than one highly qualified native speakers at Bullet Edits. All of the suggested amendments were tracked with the Microsoft Word "Track Changes" feature. Therefore, the author had the option to reject or accept each change individually.

Manuscript Title:

Network pharmacological analysis of corosolic acid reveals P4HA2 inhibits hepatocellular carcinoma progression

Anti-counterfeiting Code:

ac2cb6bb4236811d86a7b3c9aac69e34

Date Issued:

2023-03-10 22:22:22

Bullet Edits is a registered company headquartered in the UK with a global presence. We offer a range of editing, proofreading services to authors. Our Ph.D. editors are all native English speakers from the USA and UK. Authors who work with Bullet Edits are guaranteed excellent language quality and timely delivery.

BULLET EDITS LIMITED
COMPANY NO. 13345261
SEAL
★ ENGLAND AND WALES ★

Address: BULLET EDITS LIMITED, 85 Great Portland Street, London,UK
Tel: UK. (+44)20457 70286 / US.(+1)312-313-9179
Web: www.bulletedits.cn/Email: info@bulletedits.cn
VAT: GB 378 9316 43
