# Supplementary material for: Network pharmacological analysis of corosolic acid reveals P4HA2 inhibits hepatocellular carcinoma progression
Source: BMC Complement Med Ther. 2023 May 29;23:171. doi: 10.1186/s12906-023-04008-6 (PMC10226252; doi:10.1186/s12906-023-04008-6)

**Supplementary Figure S1** PPI (protein-protein interaction) network of 44 CRA targets.


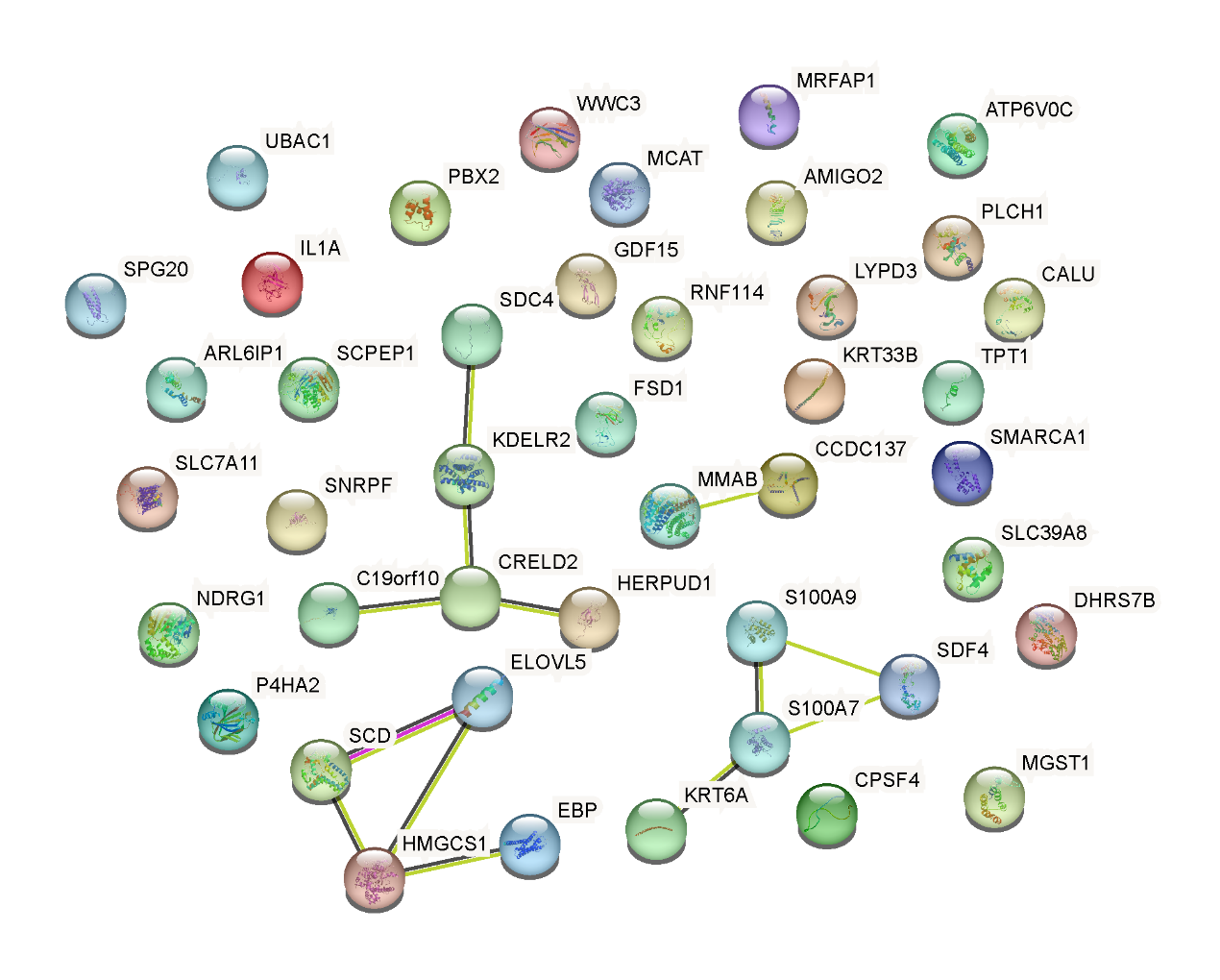


**Supplementary Figure S2** The prognostic significance of P4HA2 in LIHC analyzed by HPA database.


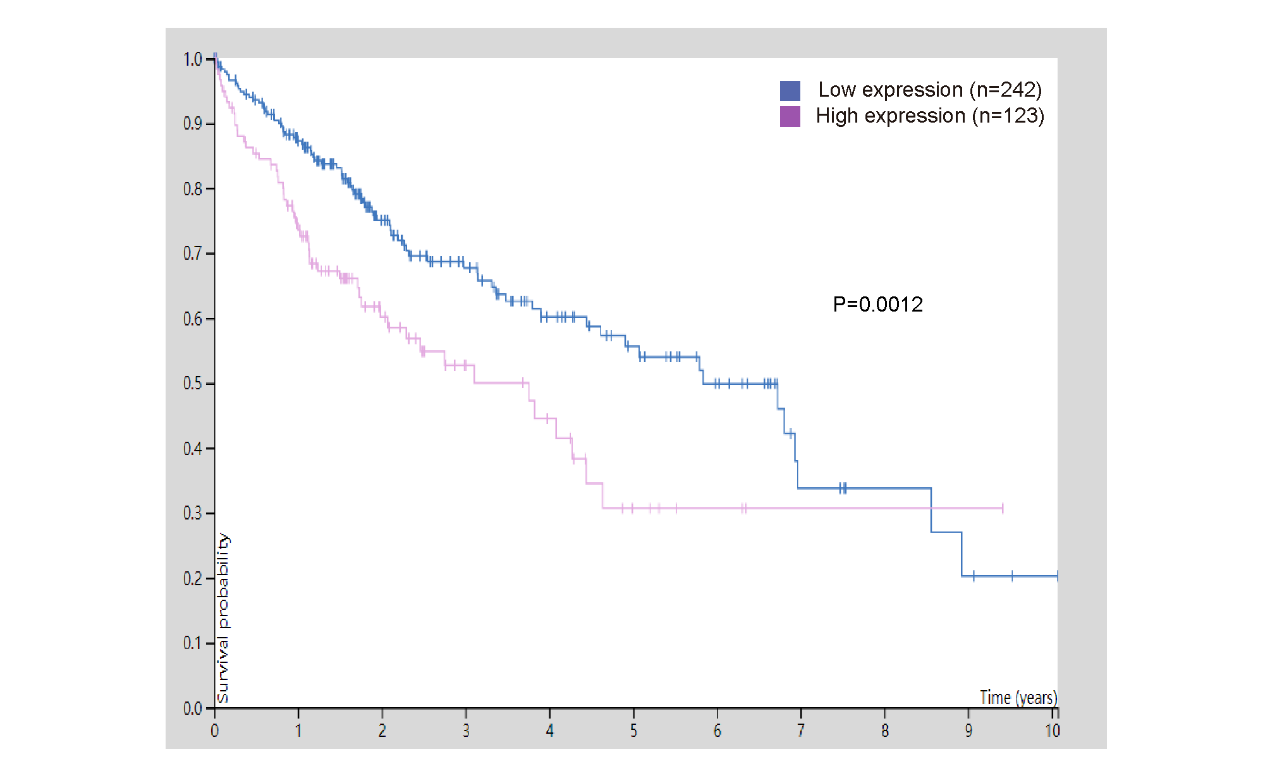

Supplement: Supplementary file 3 — Supplementary Material 3 [file 12906_2023_4008_MOESM3_ESM.docx]
